# Supplementary figures and images for: High-throughput characterization of Mycobacterium tuberculosis gene function across diverse conditions
Source: PLoS Biol. 2026 Apr 15;24(4):e3003529. doi: 10.1371/journal.pbio.3003529 (PMC13102306; doi:10.1371/journal.pbio.3003529)

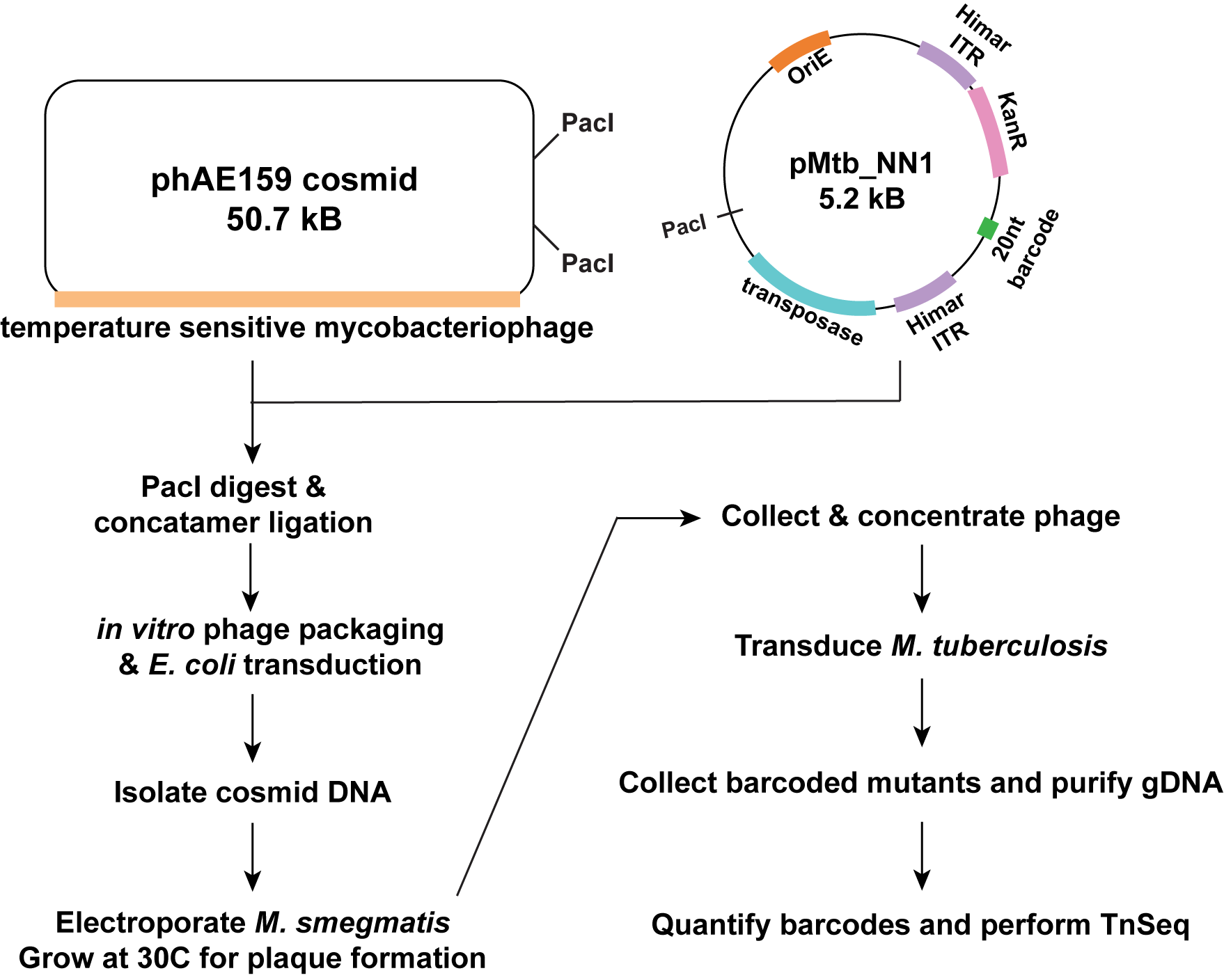

Supplement: S1 Fig — Temperature-sensitive phAE159 cosmid was combined with pMtb_NN1 containing the transposase, Himar1 mariner barcoded transposons, and a kanamycin resistant cassette via PacI digest and concatemer ligation. The ligated cosmid was packaged into lambda phage and transduced into E. coli to be midi-prepped. Barcoded phagemid was electroporated into Mycobacterium smegmatis and incubated at 30 °C for lytic phage plaque formation. Once the phage was collected and concentrated, it was transduced into Mtb and plates were incubated at 37 °C. After 21 days, colonies were scraped from plates and pooled to create the RB-TnSeq library. (TIF) [file pbio.3003529.s008.tif]

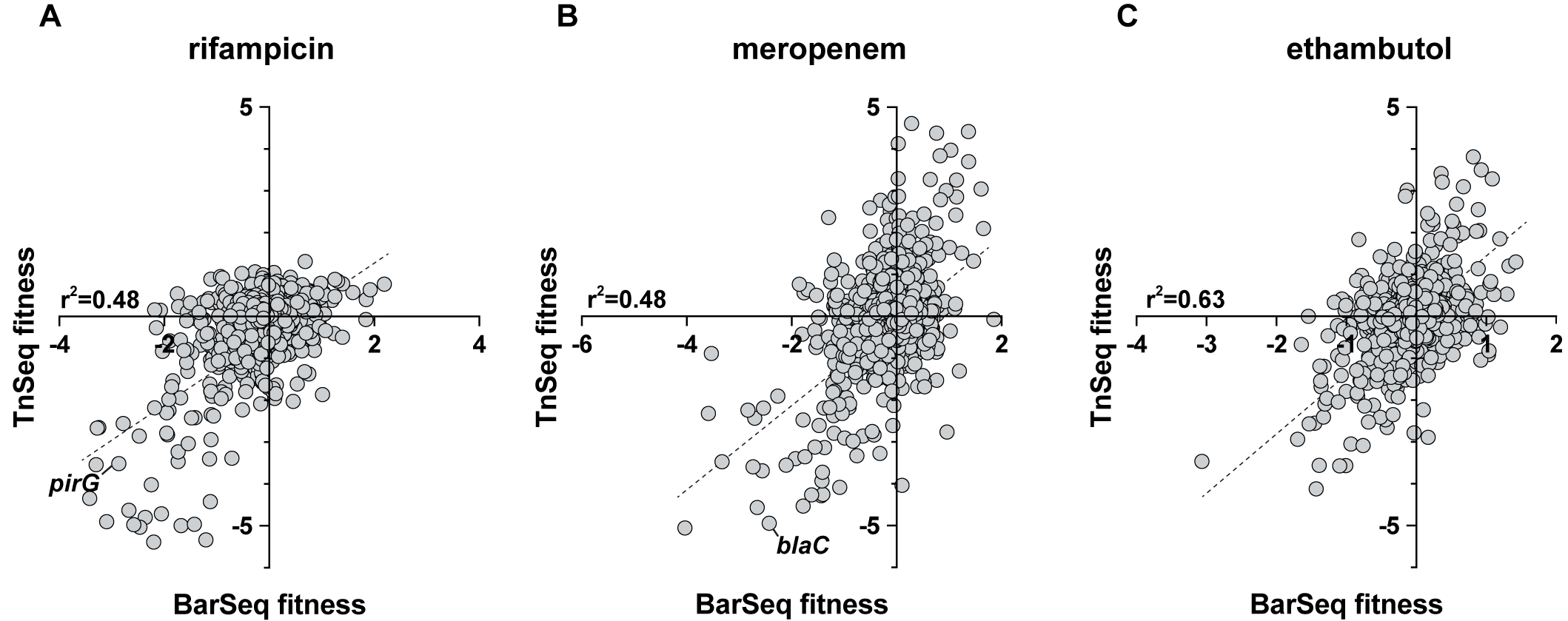

Supplement: S2 Fig — Previously published TnSeq fitness [28] plotted against RB-TnSeq fitness for rifampicin, meropenem and ethambutol. Dotted line and r2 represent linear correlation between hits with log 2 fold change >0.5 from RB-TnSeq with TnSeq. The data underlying this figure can be found in S1 Data. (TIF) [file pbio.3003529.s009.tif]

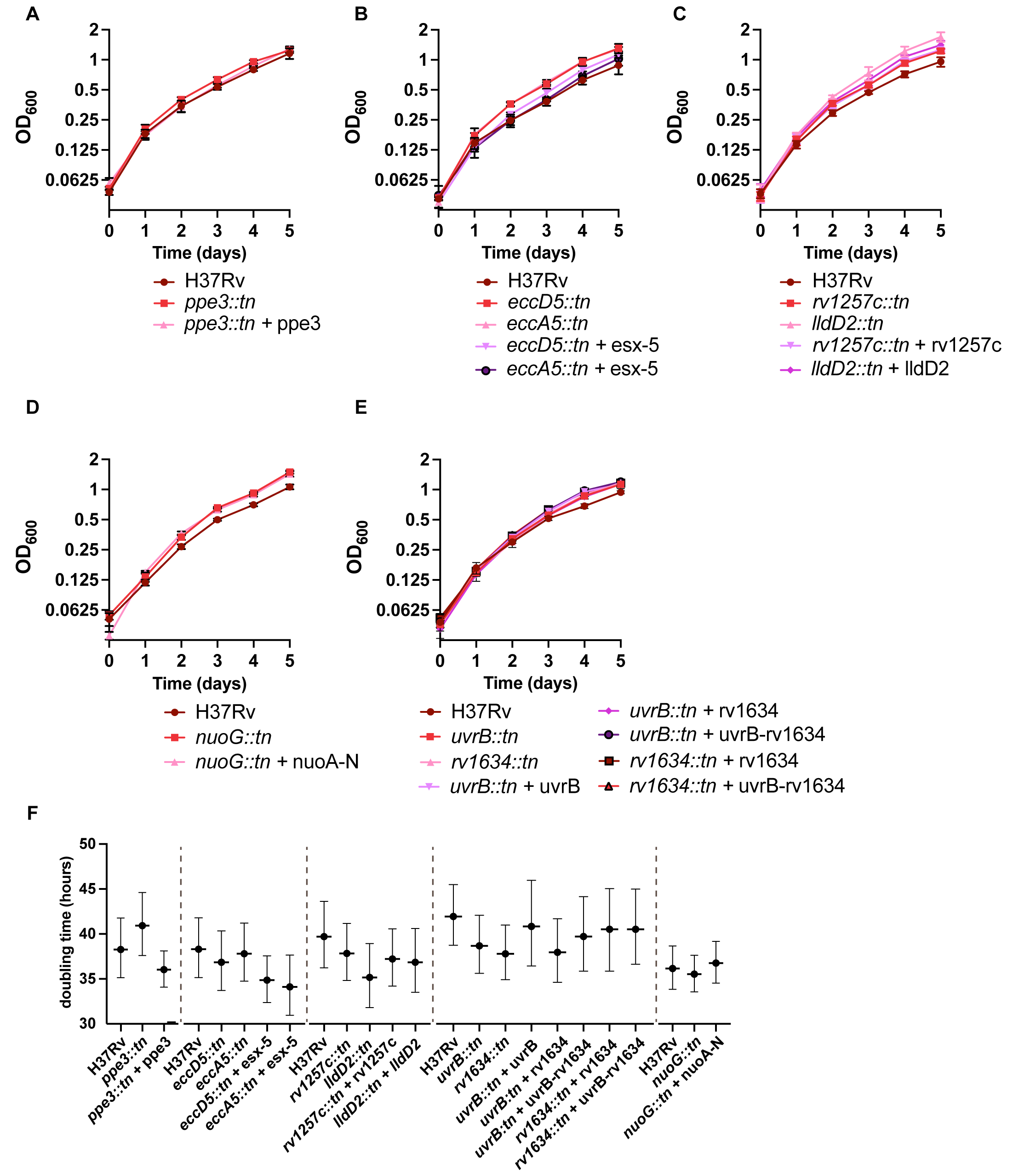

Supplement: S3 Fig — (A–E) Growth of transposon mutants and complemented strains in standard growth media (7H9) measured by OD600 over the course of 5 days. (F) Doubling time calculated from growth curves of transposon mutants and complemented strains. Data represent mean ± sd for n = 6 replicates from two independent experiments. The data underlying this figure can be found in S1 Data. (TIF) [file pbio.3003529.s010.tif]

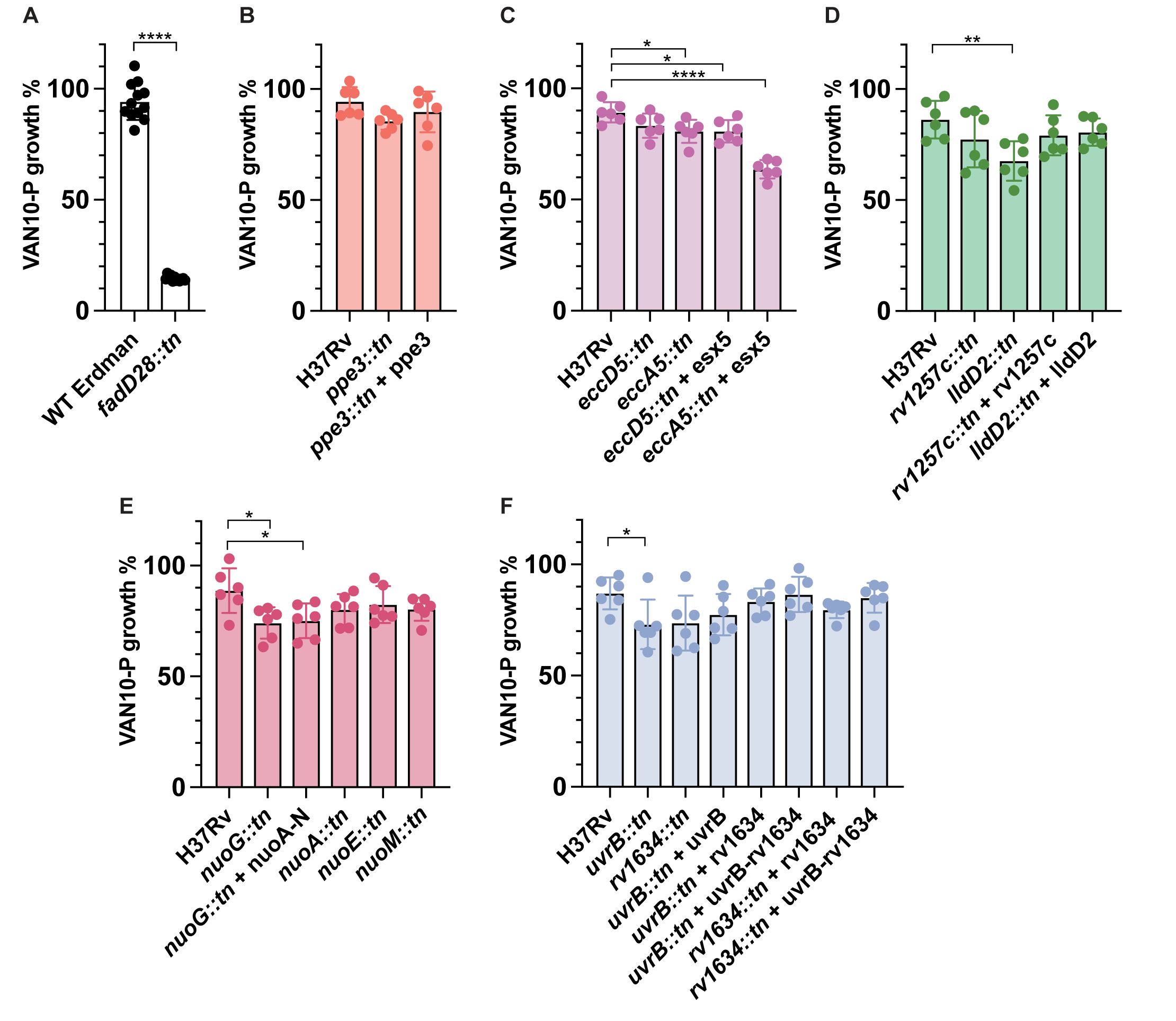

Supplement: S4 Fig — Van-10-P assay as a proxy for the PDIM levels for strains used in this study. WT Erdman and an Erdman strain lacking PDIM (fadD28::tn) are positive and negative controls, respectively. Some transposon mutant strains are slightly more susceptible to vancomycin compared to WT but are highly enriched compared to the PDIM-minus strain (fadD28::tn). Data represent mean ± sd for at least n = 6 replicates from two independent experiments. For (A), p-values were determined using Welch’s t test. For (B–F), p-values were determined using one-way ANOVA with Dunnett’s multiple comparisons test (*p < 0.05, **p < 0.01, ***p < 0.001,****p < 0.0001). The data underlying this figure can be found in S1 Data. (TIF) [file pbio.3003529.s011.tif]

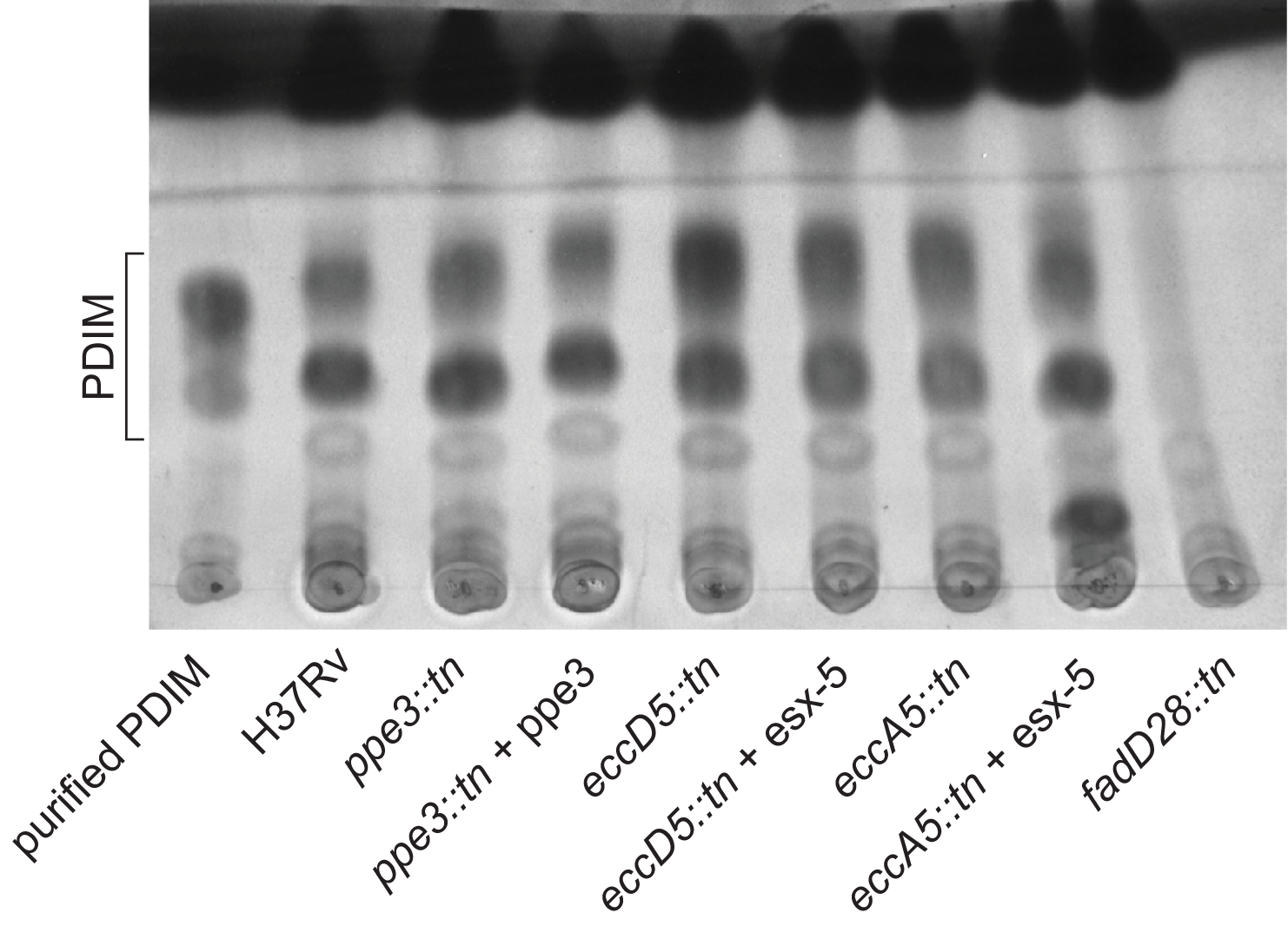

Supplement: S5 Fig — TLC lipid analysis of PDIM levels in H37Rv, ppe3::tn, eccD5::tn, eccA5::tn and complemented strains. Purified PDIM and fadD28::tn are used as positive and negative controls, respectively. TLC is representative of two biological replicates. (TIF) [file pbio.3003529.s012.tif]

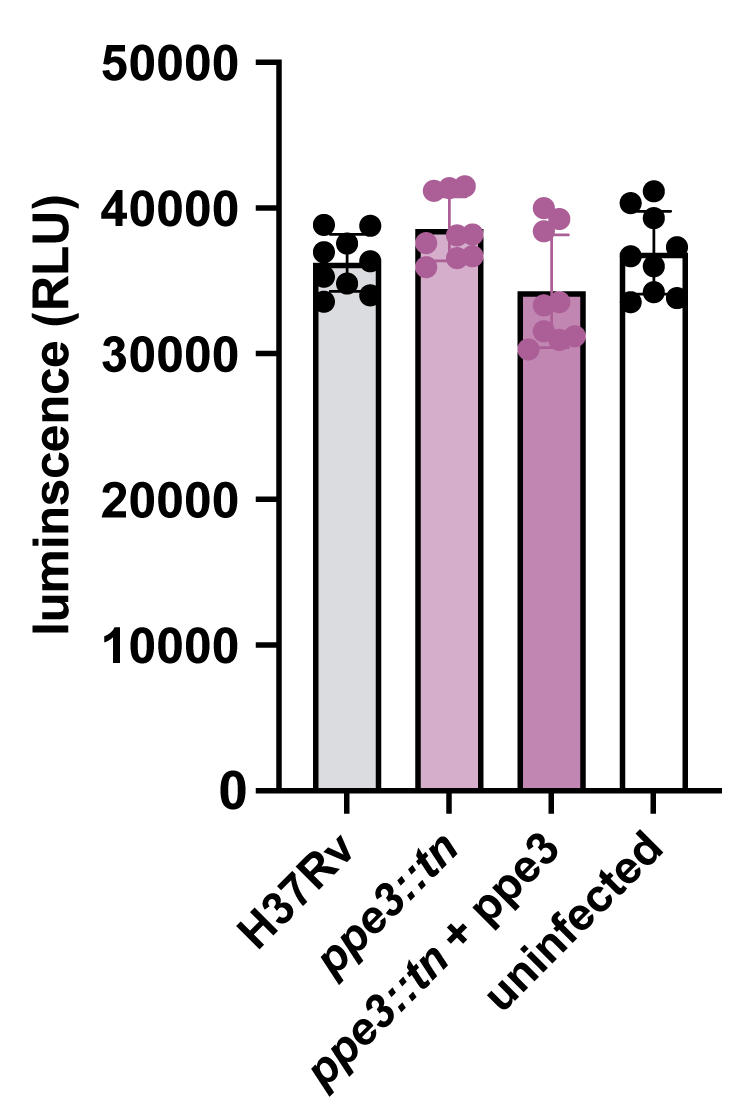

Supplement: S6 Fig — Bone marrow-derived macrophage viability measured by cell titer glo on day 4 after infection with H37Rv, ppe3::tn, and ppe3 complemented strain. Data represent mean ± sd for n = 9 replicates from three independent experiments. p-values were determined using one-way ANOVA with Dunnett’s multiple comparisons test and were all nonsignificant. The data underlying this figure can be found in S1 Data. (TIF) [file pbio.3003529.s013.tif]

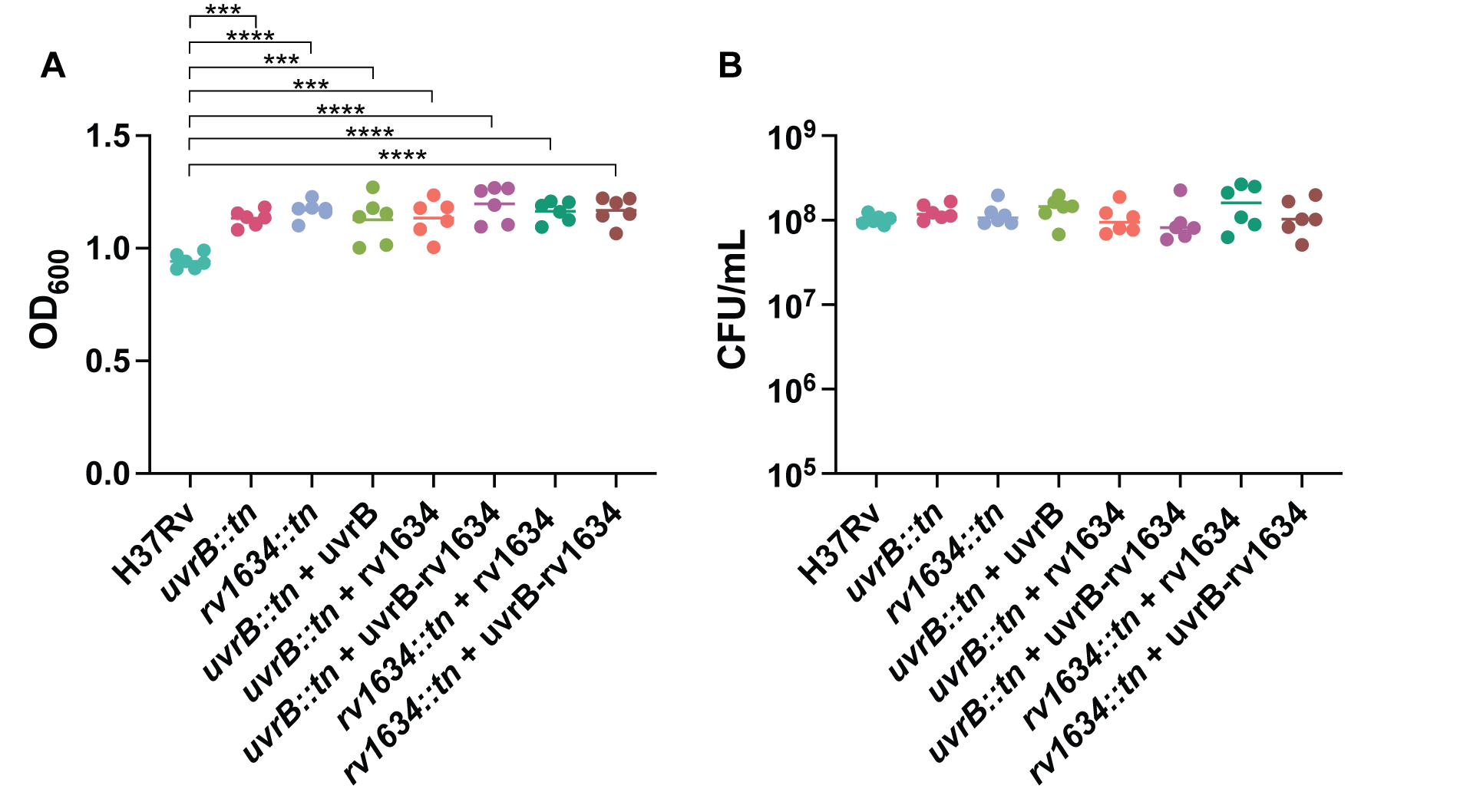

Supplement: S7 Fig — (A) OD600 and (B) CFU of untreated H37Rv, uvrB::tn, or rv1634::tn and complemented strains on day 5. Data represent mean ± sd for n = 6 replicates from two independent experiments. p-values were determined using one-way ANOVA with Tukey’s multiple comparisons test. (*p < 0.05, **p < 0.01, ***p < 0.001,****p < 0.0001). The data underlying this figure can be found in S1 Data. (TIF) [file pbio.3003529.s014.tif]
