# Supplementary material for: High-throughput characterization of Mycobacterium tuberculosis gene function across diverse conditions
Source: PLoS Biol. 2026 Apr 15;24(4):e3003529. doi: 10.1371/journal.pbio.3003529 (PMC13102306; doi:10.1371/journal.pbio.3003529)
Supplement: S1 Raw Images — (PDF) [file pbio.3003529.s016.pdf]

S5 Fig Raw Image. TLC lipid analysis of PDIM levels.

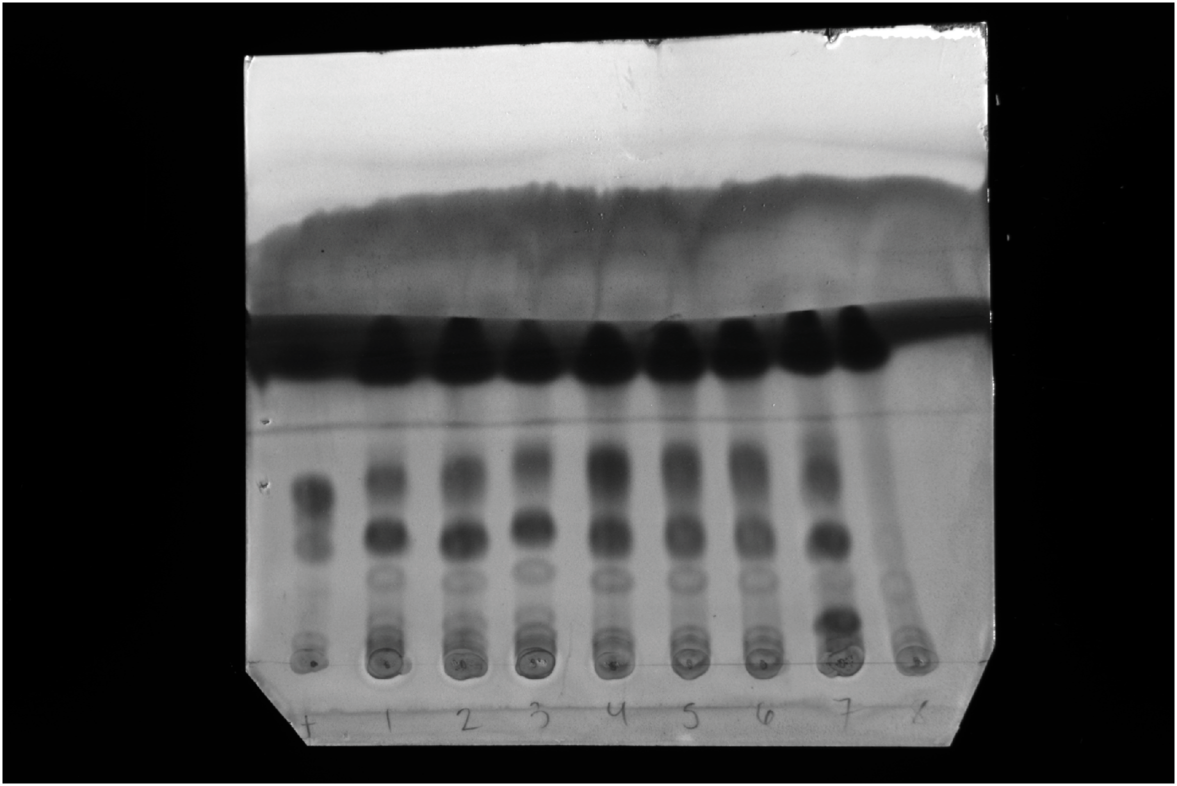

<u>Samples (from left to right):</u>

+: purified PDIM
1: H37Rv
2: *ppe3::tn*
3: *ppe3::tn* + ppe3
4: *eccD5::tn*
5: *eccD5::tn* + esx-5
6: *eccA5::tn*
7: eccA5::tn + esx-5
8: *fadD28::tn*
