# Supplementary material for: High-throughput characterization of Mycobacterium tuberculosis gene function across diverse conditions
Source: PLoS Biol. 2026 Apr 15;24(4):e3003529. doi: 10.1371/journal.pbio.3003529 (PMC13102306; doi:10.1371/journal.pbio.3003529)
Supplement: Attachment — Submitted filename: Response to reviewers.docx [file pbio.3003529.s019.docx]

**Response to Reviewers**

Reviewer #1:

The main area for improvement is in the comparison with existing datasets. The MtbTnDB database, for instance, contains more than 150 TnSeq screens, several of which were carried out under conditions similar to those tested here. It would be helpful to show how the RB‑TnSeq dataset compares with and complements these prior studies, and to what extent the new fitness and essentiality calls are consistent with previous determinations.

**We thank the reviewer for their comments. In the text, we cite the 7 previously published TnSeq screens in the MtbTnDB database that overlap with the RB-TnSeq screens (lines 221-225). We likewise compare our RB-TnSeq results to previously published TnSeq results for antibiotics in Fig 1C and S2 Fig and demonstrate that RB-TnSeq has similar results to traditional TnSeq when examining the top hits (lines 225-239).**

The authors rightly note that incomplete saturation of the RB‑TnSeq library is a "major limitation," but the manuscript does not clearly convey the magnitude of this issue. Given that M. tuberculosis gene essentiality has already been mapped by conventional TnSeq with high resolution and confidence, a direct comparison between RB‑TnSeq‑derived essentiality calls and publicly available datasets could provide an empirical estimate of false positive and false negative rates associated with the RB‑TnSeq library.

**Mapping the barcodes to their insertion site in the genome required use of traditional TnSeq. Because the unsaturation of the RB-TnSeq library arises from barcode reuse, we can estimate gene essentiality from the TnSeq data. We identify 600 essential genes, matching previous studies. This is now represented in S1 Table and the manuscript has been modified to mention essential genes (Lines 143-144). From these data, we can identify which genes are missing from the RB-TnSeq analysis due to barcode reuse. However, as mentioned, identifying gene essentiality has been previously conducted in Mtb and was not the main goal of conducting RB-TnSeq.**

----------------------------------------------------------------

Reviewer #2:

As described below, there are some points, mostly editorial in nature, that should be considered to improve the manuscript.

Major:
1.      The title "Random barcode transposon-site sequencing in Mycobacterium tuberculosis reveals the functions of uncharacterized genes" overrepresents what is provided by RB-TnSeq alone where validation was required to fully reveal functions of uncharacterized genes. It also does not capture many of the other novel genotype/phenotype associations that were discovered. I would recommend modifying the title to something involving RB-TnSeq as facilitating high-throughput elucidation of gene function across diverse conditions.

**We thank the reviewer for their suggestion. We have changed the title to “Random barcode transposon-site sequencing in *Mycobacterium tuberculosis* facilitates high-throughput characterization of gene function across diverse conditions”.**

2.      BarSeq fitness: The approach as described seems limited in its ability to provide highly reproducible fitness values between studies. The major limitation is due to not including an expansion factor, a critical experimental variable, as part of the fitness calculation. Without including population expansion as part of the calculation, "fitness" values will vary between experiments not necessarily because of actual fitness differences, but because there was a difference in the amount of growth of the population (and respective mutants). For example, say the generation time of the population is 24 hours, and a mutant of interest has a generation time of 30 hours. Using the described approach, if the population is grown for 72 hours it will expand 8-fold (log2 fold abundance change from t0 of 0), the mutant will expand 5.76 fold (log2 fold abundance change from t0 of -0.47). If the population is grown for 120 hours it will expand by 32-fold (log2 fold abundance change from
t0 of 0), the mutant will expand by 16-fold (log2 fold abundance change from t0 of -1). By not accounting for expansion, the apparent fitness of the mutant changes with the amount of growth of the population, however, in this example the mutant's fitness is the same and it is the expansion that is a variable that is unaccounted for. It is not clear whether this limitation is addressable in the current study, and if not it should be described as a limitation in the discussion in the paragraph that starts on line 461.

**We thank the reviewer for bringing up this feature of RB-TnSeq analysis. The standard analysis pipeline for RB-TnSeq does not correct the fitness values for the number of generations. The number of generations for each experiment is not variable (~3-4.5 population doublings in this study) and certain types of experiments (survival, motility, in vivo colonization, etc.) cannot easily be normalized this way. In addition, in this study and in previous RB-TnSeq studies, the data are not analyzed through comparison of magnitude of phenotypes across both genes and conditions. Lastly, in a previous study, it was demonstrated that across over 1,000 RB-TnSeq experiments and 7 bacteria that the normalization by generation time did not meaningfully impact the ability to identify significant or specific phenotypes, or cofitness calculations (see Extended Data Figure 10 in** [**https://www.nature.com/articles/s41586-018-0124-0#Sec8**](https://www.google.com/url?q=https://www.nature.com/articles/s41586-018-0124-0%23Sec8&sa=D&source=docs&ust=1770699748917741&usg=AOvVaw1Wxg5Di5xygRUwocVfNSO5)**). We have modified the manuscript under “Data Analysis” in Materials & Methods (Lines 803-808) to clarify this feature of measuring RB-TnSeq fitness.**

3.      Lines 166-168, While the statistical analysis was based on previous studies, the authors need more information and clarification on their approach and why they chose specified thresholds, which seem to differ from the cited reports which both use |t|>4 versus |t|>3 of the current study. Also, please indicate how this corresponds to threshold for FDR.

**Due to the smaller size of the Mtb RB-TnSeq, we chose to slightly relax the t-like statistic to |t|>3. We have added this statement to the text under “Data analysis” in the Materials & Methods on lines 801-803. We can approximate FDR by comparing time 0 samples to one another. Under our current thresholds, we evaluate very few statistically significant hits in the time 0 samples, suggesting a low “FDR”. Data from time 0 samples can be downloaded from the FigShare link under “Data availability” in the Materials & Methods.**

4.      There are numerous statements throughout the manuscript that confuse gene versus protein designations which detract from the flow and overall quality of the writing. Some of these errors are noted below, however, there are many more that the authors should identify and correct.
Line 117, change " a mycobacterial gene family..." to "encoding a mycobacterial protein family..."
Line 207, change "a mycothiol" to "encoding a mycothiol"
Line 209, Change "Mutants in katG (Rv1098c), a catalase-peroxidase" to "Mutations in katG (Rv1098c), encoding a catalase-peroxidase"
Line 212, Change as above.
Line 214, change "a beta-lactamase" to "encoding a beta-lactamase"
Lines 216-218, the respective statement belongs in the discussion.
Lines 223-224, change "as a" to "as encoding a" and change sugI to SugI.
Line 225, change "sugI" to "SugI" and correct all subsequent nomenclature errors where genes and proteins are not appropriately distinguished.

**We thank the reviewer for noticing the discrepancies in gene and protein nomenclature. We have modified the manuscript to the correct gene and protein designations.**

5.      Y-axis of plots in Figures 3B, C, E, F, 4B, C,  5F, G, H should be logarithmic since it will facilitate comparison of exponential growth rates. Authors should also comment on differences in growth rates and yields since carbon utilization is a major aspect of the study.

**We thank the reviewer for their suggestion, and we have changed the y-axis of these graphs to a log2 scale to highlight the exponential growth rates and yields of the transposon mutants on their given carbon source.**

Minor:
Lines 77-80, Stating patient non-adherence as the driving factor for the rise in MDR- and XDR-TB is incomplete and can be viewed as offensive to some. I recommend modifying "resulting in patient non-adherence" to "contributing to treatment disruption".

Lines 91-92, It is true that there are many unannotated genes (as yet unidentified open reading frames) in Mtb, however, those genes with the annotation of "hypothetical protein" are indeed annotated. Unannotated genes were not assessed in the described approach since the approach was dependent upon gene annotation. As such, it would be better to say that these "genes remain uncharacterized with many being annotated as encoding hypothetical proteins and others with functional annotations based on homology with orthologs from other species."

**We thank the reviewer for these suggestions which we have completed.**

Lines 92-94, It is not clear how points in this sentence fit with the story and they were not address, I recommend deleting.

**Done.**

Line 98, "infer" is an over-representation of what is learned by TnSeq. Change "infer" to "better understand", or something of the like.

**Done.**

Line 124, change "for drug resistant Mtb strains." to "for treatment of drug resistant Mtb infections."

**Done.**

Lines 141-143, These statements on difficulty, loss of diversity at each step and optimization refer to data that is not shown. Please either include as supplemental data or omit this statement.

**Reviewer 4 suggested including additional information about the library construction optimization. Thus, we have modified the text to include more details in the discussion (lines 535-540) and materials & methods (lines 665-669) to clarify what optimization steps were made.**

Lines 163-164, Modify "Mutants in genes..." to "Bar codes associated with a growth advantage..." or something of the like.

**Done.**

Line 171, change "were" to "involved"

**Done.**

Line 184, change "conducted" to "administered", or something of the like

**Done.**

Line 312, Figure S4 does not show testing of all strains in the VAN-P-10 assay. Please modify statement accordingly. Also, since PDIMs were not directly measured, it would be best to say "likely retain PDIM".

**Done.**

Line 415, Change "Mutants" to "Mutations"

**Done.**

Line 441-447, should move this to discussion.

**Done.**

Line 452, "This high-throughput data allows" to "These high-throughput allow"

**Done.**

Line 508, change "mutant" to "mutation"

**Done.**

Line 510-511, An alternative possibility is that in the absence of Rv1634 function, a different efflux or other drug tolerance mechanism could be induced.

**We thank the reviewer for this idea. We have added it to the manuscript (line 593-594)**

Line 510, change "mutant" to "mutation"

**Done.**

Line 535, Was a different iron salt used? If not, phenotypes could be associated with a change in iron abundance.

**While there could technically be a difference in iron abundance, it is difficult to see differences even in iron related phenotypes (such as siderophores) without explicit removal of iron using chelators.**

Line 581, change "conicals" to "conical tubes"

**Done.**

Line 595, change "dissolved" to "disbursed"

**Done.**

----------------------------------------------------------------

Reviewer #3:

Major suggestions

Given the reported issues with loss of cell surface PDIM and how this confounds measurements of PPE associated functions in Mtb (Reference #41) and infection studies, I suggest you use a second method to confirm that your WT, ppe3::tn, eccA5::tn, and complemented strains do actually produce PDIM.  I suggest this because the ppe3 and eccA5 mutants have a pronounced growth defect in propionate media (Fig 3 and S6 table) suggesting an issue in propionate uptake/metabolism. This is especially important since the Van-10-P assay used to predict the presence of PDIM in your strains (S4 Figure) is dependent on normal levels of propionate import/metabolism by the bacteria.

**We agree with the reviewer that the van-10-P assay may be affected by these Mtb mutants. We have performed thin layer chromatography (TLC) lipid analysis of PDIM to verify that the *ppe3* and *esx-5* mutants and their complemented strains produce PDIM (S5 Fig).**

It Is not demonstrated in this manuscript that ESX-5 secretes PPE3 (lines 454-455). The lactate metabolism defect in the ESX-5 mutants could be explained by the loss of another PPE protein from the bacteria's surface.

Can you provide evidence that PPE3 is secreted in an ESX-5 dependent manner or is this published somewhere?

**This is the work of future studies. We have reworded the manuscript to account for the reviewer’s comment.**

Minor suggestions

I think line 157 should read "131 stressors and antibiotics" to match data in Table S3. Similar on line 183?

**We have edited the text to be more clear about the numbers of experiments and how this corresponds to the number of replicates, conditions, and compounds tested.**

The numbers on line 193 do not seem to match the supplement data, it looks like 89 unique conditions were screened (20 carbon, 10 nitrogen, 59 stress). Please double check this. Maybe explain where the 212 number came from, did some conditions have more replicates?

**See above.**

Line 200, should this read 89 instead of 95?

**See above.**

Line 450, make this consistent with the condition #'s above if it is not accurate.

**See above.**

Line 484, lldD1 has previously been flagged as being required for cholesterol utilization via tnSeq (PMID: 22365605).

**We thank the reviewer for pointing this out and have cited the paper in reference to lldD1.**

----------------------------------------------------------------

Reviewer #4:

My primary concern is that, while the authors emphasize the benefits of RB-TnSeq in the Introduction, they also state that library generation presented considerable difficulty. However, these difficulties are not described in sufficient detail in the main text, which may limit the ability of other labs to adopt this approach. The authors note that "significant optimization" was required, but do not elaborate on what aspects of the workflow were challenging or how these challenges were overcome.

**We thank the reviewer for their suggestion to elaborate on the RB-TnSeq library construction. We have added more description to the discussion (lines 535-540) and materials & methods (lines 665-669) to clarify what optimization steps were made.**

Relatedly, the Discussion focuses almost exclusively on the biological findings, and does not address the advantages and disadvantages of RB-TnSeq relative to other functional genomics approaches. For example, how does library generation compare to conventional TnSeq in M. tuberculosis? Why do the authors think it was particularly difficult to generate or maintain library diversity? How does RB-TnSeq compare to CRISPRi-based screens in terms of scalability, coverage, or experimental limitations? While I appreciate the biological insights presented, this omission feels like a missed opportunity. As this study represents the first proof-of-principle application of RB-TnSeq in M. tuberculosis, a more explicit discussion of the technical challenges, trade-offs, and future optimization would help set the stage for broader adoption of this promising approach.

**We have modified the discussion to include more details about difficulty constructing the library and how RB-TnSeq compares to other approaches such as CRISPRi (lines 528-551). We thank the reviewer these points and improving the discussion.**

Minor
Lines 159: Is saturation more than 3 doubling as stated in the paragraph above?

**We have edited the text to clarify.**

Figure 2 - the text is much too small to be legible

**We have increased the font size in Figure 2.**
