# Supplementary material for: High-throughput characterization of Mycobacterium tuberculosis gene function across diverse conditions
Source: PLoS Biol. 2026 Apr 15;24(4):e3003529. doi: 10.1371/journal.pbio.3003529 (PMC13102306; doi:10.1371/journal.pbio.3003529)
Supplement: Attachment — Submitted filename: Response to Reviewers2.docx [file pbio.3003529.s020.docx]

Reviewer #1:

I fully support acceptance of the revised manuscript.

-----------------------------------------------------------------------


Reviewer #2:

The authors have done a nice job of addressing comments raised in review. My only additional suggestions is to change "genetic approaches" to "mutation-based genetic approaches" on line 28 of the abstract. The current statement ignores the use of CRISPRi as a large-scale genetic approach to understand gene function.

**We thank the reviewer for their suggestion and have edited the text.**
-----------------------------------------------------------------------


Reviewer #3:

The comments I made in the previous submission have been addressed. The authors double checked the sections that previously did not seem consistent in this version.

I have no more major suggestions to make.

-----------------------------------------------------------------------


Reviewer #4:

This manuscript has been significantly improved. I have no further suggestions.
